# Neuroprotection in acute brain injury: an up-to-date review

**DOI:** 10.1186/s13054-015-0887-8

**Published:** 2015-04-21

**Authors:** Nino Stocchetti, Fabio S Taccone, Giuseppe Citerio, Paul E Pepe, Peter D Le Roux, Mauro Oddo, Kees H Polderman, Robert D Stevens, William Barsan, Andrew IR Maas, Geert Meyfroidt, Michael J Bell, Robert Silbergleit, Paul M Vespa, Alan I Faden, Raimund Helbok, Samuel Tisherman, Elisa R Zanier, Terence Valenzuela, Julia Wendon, David K Menon, Jean-Louis Vincent

**Affiliations:** Department of Physiopathology and Transplant, Milan University, Fondazione IRCCS Cà Granda Ospedale Maggiore Policlinico, Via F Sforza 35, 20122 Milan, Italy; Neuro ICU, Fondazione IRCCS Cà Granda Ospedale Maggiore Policlinico, Via F Sforza 35, 20122 Milan, Italy; Department of Intensive Care, Erasme Hospital, Université libre de Bruxelles, 1070 Brussels, Belgium; Department of Health Science, University of Milan-Bicocca, Ospedale San Gerardo, Via Pergolesi 33, 20900 Monza, MB Italy; NeuroIntensive Care, Department of Emergency and Intensive Care, Ospedale San Gerardo, Via Pergolesi 33, 20900 Monza, MB Italy; Department of Emergency Medicine, University of Texas Southwestern Medical Center at Dallas, Dallas, TX 75390-8579 USA; Department of Neurosurgery, Lankenau Medical Center, Wynnewood, PA 19096 USA; Department of Intensive Care Medicine, Centre Hospitalier Universitaire Vaudois (CHUV) University Hospital, Faculty of Biology and Medicine, University of Lausanne, 1011 Lausanne, Switzerland; Department of Critical Care Medicine, University of Pittsburgh Medical Center, Pittsburgh, PA 15213 USA; Division of Neuroscience Critical Care, Division of Adult Critical Care, Johns Hopkins Medicine, Baltimore, MD 21287 USA; NETT Clinical Coordinating Center, University of Michigan, Ann Arbor, MI 48106-0381 USA; Department of Neurosurgery, University Hospital Antwerp and University of Antwerp, 2650 Edegem, Belgium; Department of Intensive Care, UZ Leuven, Campus Gasthuisberg, 3000 Leuven, Belgium; Department of Critical Care Medicine and the Safar Center for Resuscitation Research, University of Pittsburgh Medical Center, Pittsburgh, PA 15213 USA; Department of Neurocritical Care, David Geffen School of Medicine at UCLA, Los Angeles, CA 90095 USA; Center for Shock, Trauma & Anesthesiology Research (STAR), University of Maryland School of Medicine, Baltimore, MD 21201 USA; Department of Neurology, Neurocritical Care Unit, Innsbruck Medical University, 6020 Innsbruck, Austria; Department of Surgery, R Adams Cowley Shock Trauma Center, University of Maryland School of Medicine, Baltimore, MD 21201 USA; IRCCS – Istituto di Ricerche Farmacologiche Mario Negri, Department of Neuroscience, 20156 Milan, Italy; Department of Emergency Medicine, University of Arizona, Tucson, AZ 85724-5057 USA; Institute of Liver Studies, King’s College Hospital, Denmark Hill, London, SE5 9RS UK; Division of Anaesthesia, University of Cambridge, Addenbrooke’s Hospital, Cambridge, CB2 0QQ UK

## Abstract

Neuroprotective strategies that limit secondary tissue loss and/or improve functional outcomes have been identified in multiple animal models of ischemic, hemorrhagic, traumatic and nontraumatic cerebral lesions. However, use of these potential interventions in human randomized controlled studies has generally given disappointing results. In this paper, we summarize the current status in terms of neuroprotective strategies, both in the immediate and later stages of acute brain injury in adults. We also review potential new strategies and highlight areas for future research.

## Introduction

Acute brain injury, whatever its cause, is associated with considerable short-term and long-term morbidity and mortality. In the USA it is estimated that 52,000 fatalities arise as a result of traumatic brain injury (TBI) every year, and approximately 5.3 million people live with TBI-related disabilities [1]. These figures are similar in the European Union, where an estimated 7.7 million people have TBI-related disabilities [2]. Accurate data from emerging economies, where TBI is increasing due to greater motorization, are lacking, but are likely to be similar or worse [3]. Stroke is the second leading cause of death and the third leading cause of disability-adjusted life-years worldwide, and its global burden is increasing [4]. Other ischemic and hemorrhagic lesions to the brain, such as subarachnoid hemorrhage or ischemia–reperfusion after cardiac arrest, are also associated with high mortality and devastating sequelae [5,6].

Following the primary cerebral insult, a cascade of events amplifies the initial damage regardless of the etiology of the precipitating event. Secondary biochemical changes contribute to subsequent tissue damage with associated neuronal cell death. The time course over which these effects occur may be longer than assumed previously, potentially providing a wider time window for interventions. Neuroprotective agents that can limit secondary tissue loss and/or improve behavioral outcomes have been identified in multiple animal models of acute brain injury. However, translation to the clinical setting has been largely disappointing. Box 1 summarizes important methodological issues in animal studies that have often not been adequately addressed before clinical trial.

Given the severity and long-term consequences and costs of brain damage from many different etiologies, strategies for neuroprotection are of obvious importance. In this review, based on expert opinion and nonsystematic literature review, we provide an up-to-date summary of the current evidence for strategies to protect the brain from secondary insults and highlight areas for future experimental and clinical research. We have focused on general concepts in selected pathophysiological events, because complete coverage of all acute brain injuries is not feasible in one manuscript, and we restricted our analysis to adult patients.

## Existing therapeutic modalities for neuroprotection

### Reperfusion strategies in ischemic stroke

Early reperfusion therapy to restore blood flow to salvageable ischemic brain can prevent cell death and facilitate neurological recovery. Reperfusion may be accomplished through administration of intravenous thrombolytic agents, intra-arterial thrombolysis, mechanical thromboembolectomy, ultrasound-enhanced thrombolysis and various combinations of these approaches. Alternative or adjunct approaches include enhanced oxygen delivery, hemodilution and systemic central hemodynamic augmentation therapy [7]. Risks associated with reperfusion therapies, apart from failure to reperfuse, include intracerebral hemorrhage (ICH), ischemia–reperfusion injury and catheterization complications. A large-scale randomized trial indicated that intravenous recombinant tissue plasminogen activator (alteplase) administered within 3 hours of ischemic stroke onset was associated with significantly improved functional outcomes when compared with placebo, but with an increased risk of ICH [8]. More recent data indicated that intravenous alteplase was beneficial when given within 4.5 hours of onset in nondiabetic patients <80 years old without massive strokes [9].

Despite increased risks of ICH and early death, a Cochrane review of 27 controlled trials of thrombolytic agents given within 6 hours of acute ischemic stroke onset indicated improved survival and neurological outcome at 3 to 6 months; benefits were greater in patients treated within 3 hours [10]. The evidence offered by these papers has been incorporated into guidelines, which recommend early intravenous thrombolysis [11].

There is ongoing interest in selectively treating patients who have a mismatch between brain tissue that is hypoperfused on neuroimaging but deemed salvageable and tissue that has or is predicted to infarct, because studies have demonstrated that patients who have a mismatch are more likely to have a favorable outcome following reperfusion than those who do not [12,13]. Restoring perfusion by selectively targeting the occluded artery via intra-arterial thrombolysis or mechanical thromboembolectomy may also be beneficial in patients with ischemic stroke symptom onset of >4.5 hours or those for whom systemic intravenous thrombolysis is contraindicated [11,14]. Recent data on intra-arterial strategies as an alternative or supplement to intravenous thrombolysis in tissue plasminogen activator-eligible patients have been mixed [13,15-17], but they indicate potential benefit in stroke with proximal large vessel occlusions.

### Prevention of secondary insults after traumatic brain injury

Various insults can aggravate the initial traumatic brain damage and preventing or minimizing such insults represents a form of brain protection. For example, early removal of large intracranial hematomas that compress the brain is a key intervention. However, published supportive evidence is lacking and randomized trials comparing early versus delayed surgery would probably encounter ethical challenges [18]. Nevertheless, in the case of epidural hematoma management, shortening the time from trauma to surgery was associated with improved outcomes in a prospective cohort study [19]. Decompressive craniectomy is sometimes used in TBI patients with raised intracranial pressure (ICP) not responding to first-tier intensive care and neurosurgical therapies. A randomized controlled trial, however, found that those randomized to decompressive craniectomy had decreased ICP and length of ICU stay but more unfavorable outcomes as determined by the Extended Glasgow Outcome Scale at 6 months after injury [20].

The deleterious effects of hypoxia and arterial hypotension in the early phases after TBI have been identified in several analyses. Data from the US Traumatic Coma Data Bank collected from 1983 to 1988 showed that among 717 TBI admissions, hypoxia (defined as arterial oxygen tension <60 mmHg, cyanosis or apnea) and/or hypotension (defined as systolic pressure <90 mmHg) were identified in 45% of cases and were independently associated with significant increases in morbidity and mortality [21]. Similar findings were reported in the International Mission on Prognosis and Analysis of randomized Controlled Trials (IMPACT) in TBI study [22] and confirmed in other recent reports [23,24]. Correction of arterial hypotension and hypoxia is therefore mandatory [25].

There is no evidence to indicate the superiority of new approaches, such as hypertonic fluids, over conventional isotonic saline to correct hypotension [26]. The role of prehospital intubation in neuroprotection has been questioned in the past [27], but more recent studies suggest it may be associated with more favorable outcomes, as evaluated by the Extended Glasgow Outcome Scale score at 6 months [28]. Increased ICP, reduced cerebral perfusion, seizures, and so forth, represent further secondary insults to the traumatized brain; early detection and prompt, intensive treatment may contribute to better outcomes [29].

### Autoregulation and neuroprotection

Cerebrovascular autoregulation is the ability of the brain to maintain a constant cerebral blood flow (CBF) through a range of cerebral perfusion pressures (CPP) [30]. Several dynamic pressure reactivity indices have been proposed for monitoring cerebrovascular autoregulation in real time at the bedside, by calculating the correlation between the arterial blood pressure and continuous measures of CBF or cerebral blood volume [30]. When this correlation is negative or close to zero, autoregulation is assumed to be present (pressure active); when it is positive, autoregulation is considered to be absent (pressure passive). ICP, transcranial Doppler, brain tissue oxygenation and near-infrared spectroscopy have all been used as estimates of global CBF/cerebral blood volume. Accordingly, the CPP range at which autoregulation is best preserved (optimal CPP) can be estimated. However, whether a strategy based on these measurements might be used to improve outcomes has yet to be demonstrated [31].

### Hemoglobin management for neuroprotection

Anemia is common among patients with severe brain injury and is associated with poor outcomes in TBI, aneurysmal subarachnoid hemorrhage (SAH), ICH and acute ischemic stroke [32,33]. In the absence of serious cardiac disease, a restrictive red blood cell transfusion strategy (for example, trigger hemoglobin (Hb) 7 g/dl) is usually recommended in critically ill patients [34]. In severe brain injury, however, compromised brain tissue oxygenation may occur at higher Hb levels than in other ICU patients [35,36].

In a recent randomized clinical trial in 200 patients with closed head injury, erythropoietin was compared with placebo in patients with transfusion thresholds of 7 g/dl versus 10 g/dl [37]. There were no statistically significant differences in 6-month neurological outcomes between the two transfusion groups, but the higher transfusion threshold was associated with more adverse events. Based on these limited numbers of patients, it seems premature to conclude that a low transfusion threshold may be beneficial in all TBI patients; more data are needed.

In a pilot trial involving 44 aneurysmal SAH patients with high risk of cerebral vasospasm, Naidech and colleagues reported no significant difference in the predefined safety endpoints when using a liberal transfusion strategy to maintain Hb >11.5 g/dl compared with a conservative approach (target Hb >10 g/dl) [38]. In a *post hoc* analysis, however, patients in the lower Hb group had more cortical infarctions, suggesting that higher Hb thresholds may be needed to provide protection from vasospasm-related infarction. In one retrospective analysis of 205 consecutive patients with aneurysmal SAH, there was a higher risk of thrombosis, pulmonary embolism and poor outcome in patients receiving blood transfusion [39]. This correlation persisted in multivariable analysis: odds ratio of 2.4 (95% confidence interval = 1.2 to 4.6, *P* = 0.01) for thromboembolic event, and odds ratio of 5.0 (95% confidence interval = 1.9 to 12.8, *P* <0.01) for poor outcome.

TBI and aneurysmal SAH are clearly distinct entities and require specific studies to identify optimal Hb levels. In both pathologies, however, the benefits of red blood cell transfusion may only exceed the associated risks if physiologic triggers – for example, brain tissue hypoxia or brain metabolic distress – indicate that transfusion is required. In this context, simultaneous occurrence of anemia and compromised brain tissue oxygen tension (PbtO_2_), but not anemia alone, was correlated with poor 1-month outcomes in patients with TBI [40].

### Preserving brain perfusion in sepsis

Brain dysfunction is frequent during sepsis and is associated with increased mortality and long-term cognitive dysfunction. The pathophysiology is complex and poorly understood [41], but decreased brain perfusion may be a major determinant [42]. Some studies have shown lower CBF in septic patients than in healthy volunteers, but other factors, such as sedative agents or hypocapnia associated with hyperventilation, may also explain the CBF reduction in such patients. Sepsis is also associated with impaired CBF autoregulation, however, especially when shock is present [42,43]. A low arterial carbon dioxide tension may enhance or restore the regulation of brain perfusion in septic patients, without significantly affecting cerebral oxygen metabolism [44]. However, the impact of hypocapnia in these patients needs to be studied in larger cohorts. Brain dysfunction during sepsis also occurs in hemodynamically stable patients and experimental data have suggested that regional brain perfusion can become inadequate because of microcirculatory alterations [45].

An optimal threshold of blood pressure (or any other metric) to prevent brain hypoperfusion in septic patients has not yet been identified. Novel monitoring tools will hopefully help improve our understanding of CBF and microvascular regulation and facilitate identification of specific neuroprotective strategies to minimize secondary ischemic brain injuries in this setting.

### Hepatic encephalopathy

Etiological factors in hepatic encephalopathy relate largely to increased circulating ammonia with changes in cellular glutamine/glutamate levels, altered neurotransmitter signaling and subsequent astrocyte swelling, and microglial and mitochondrial dysfunction [46]. Drugs to decrease arterial ammonia levels have not been shown to be effective in acute liver failure but may have a role in cirrhosis. The use of novel agents to increase skeletal metabolism of ammonia has been shown to be effective in animal models [47]. Ammonia levels may also be manipulated by renal replacement therapies. Control of the inflammatory phenotype may also be relevant, with animal studies showing that decreasing brain inflammation may be beneficial. Clinical studies in acute liver failure suggest that combined control of ammonia (with renal replacement therapy), inflammatory phenotype, temperature and hyponatremia may be beneficial [48,49]. The long-term effects of hepatic encephalopathy in patients with acute liver failure have not been widely examined but those who recover are largely neurologically intact. In those individuals with cirrhosis, chronic encephalopathy can be seen and may be difficult to distinguish from other neurological syndromes. Liver transplantation largely results in resolution of encephalopathy.

### Body temperature, brain temperature and neuroprotection

Brain temperature has been shown to exceed measured core temperature by between 1 and 2°C in various types of brain injury [50]. Numerous studies in patients with acute brain injury have shown links between fever and adverse outcome, regardless of the cause of fever [51]. Some studies have shown links between low-grade hyperthermia (>37.5°C) and adverse outcomes [52]; however, evidence that treating fever improves outcomes is still lacking.

Controlled lowering of core body temperature to mitigate secondary injuries (including reperfusion injury) after acute brain injury has been widely studied. Multiple animal studies have shown that therapeutic (induced) hypothermia (TH) may mitigate ischemia–reperfusion injury and reduce brain edema. Both mechanisms may be present within the same patient, but the two processes differ and, crucially, have different time frames requiring fundamentally different approaches to treatment. Ischemia–reperfusion involves mechanisms that begin within minutes to hours after injury, and effects last up to 72 hours after the initial ischemic insult (with the important exception of apoptosis, which starts later and may be more prolonged) [52,53]. Therefore, at least theoretically, ischemia–reperfusion requires rapid treatment, ideally before or in the early stages of injury and lasting up to 72 hours as an optimal time frame. In contrast, brain edema would require treatment for as long as it persists, and thus treatment periods would be highly variable.

Clinical studies attempting to translate the promising animal TH data to the bedside have yielded variable and sometimes conflicting results. There is strong evidence from studies in TBI, acute ischemic stroke with brain edema and acute hepatic encephalopathy that use of TH lowers ICP. However, reduced ICP does not necessarily equate with improved outcome, and clinical results have been variable. Most studies using TH for limited durations in severe TBI have not found benefits for neurological outcome, and studies involving TH for limited periods according to a fixed protocol often reported rebound increases in ICP when patients were rewarmed. Better results have been reported from studies using TH for more prolonged periods (4 to 5 days) [51]. A large European trial testing the use of TH to control ICP (Current Controlled Trials ISRCTN34555414) has been recently closed to recruitment, with no data yet available on outcomes. An Australian–New Zealand study (Clinical Trials NCT00987688) of very early TH to improve outcome after severe TBI is currently ongoing.

Hypothermia in acute ischemic stroke was initially used to control malignant brain edema in patients with large middle cerebral artery stroke [51]. More recently, pilot studies have used hypothermia to mitigate ischemia–reperfusion injury in patients with acute ischemic stroke undergoing thrombolysis or mechanical thrombectomy. Of note, these studies involved nonintubated and only mildly sedated patients, and used TH strategies such as skin counterwarming, magnesium drips and buspirone administration to manage shivering. The results are promising but as yet inconclusive.

Following out-of-hospital cardiac arrest, recent data suggest that very mild hypothermia (36°C) may be as protective as moderate hypothermia (33°C) [54], although this remains a topic of debate [55]. Patients with post-traumatic cardiac arrest have a grim prognosis. Surgeons often cannot stop bleeding quickly enough to prevent irreversible damage to the brain and other organs, even if the original injuries are technically reparable. Emergency preservation and resuscitation may represent a method for preserving life during a period of no blood flow to allow time for the surgeon to achieve hemostasis, followed by delayed resuscitation using cardiopulmonary bypass. Currently, the best approach to inducing emergency preservation and resuscitation is an intra-aortic flush of ice-cold saline to achieve a brain temperature of 10°C. In animal models, this profound cooling has allowed survival with good neurologic outcome after prolonged hemorrhage leading to cardiac arrest [56] or rapid exsanguination followed by 2 to 3 hours of circulatory arrest [57]. A clinical safety and feasibility trial is underway in the USA in patients with penetrating trauma leading to cardiac arrest within 5 minutes of emergency department arrival or in the emergency department (Clinical Trials NCT01042015).

## Novel therapeutic modalities

### Neurorepair strategies

In addition to neurological damage, acute brain injury induces a series of neurorestorative events [58]. In some cases, the central nervous system is able to remodel itself following insults that impair tissue homeostasis. Neurorestorative events include neurogenesis, gliogenesis, angiogenesis, synaptic plasticity and axonal sprouting. These processes are stimulated by endogenous growth-related factors and may continue for weeks to months, facilitating functional and structural recovery. Unfortunately, these restorative processes are largely ineffective for the severity of damage usually encountered in TBI or stroke. Accordingly, providing such injured tissue with a milieu that enhances neuroregenerative processes has become an important therapeutic target.

### Mesenchymal stromal cells

Infusion of mesenchymal stromal cells (MSCs) can improve structural and functional outcomes in different brain injury models [59]. MSCs secrete growth and neurotrophic factors or induce their production by resident brain cells, including microglia. The interaction between MSCs and inflammatory microenvironments is crucial. MSCs can reprogram the local microenvironment from a detrimental function to a beneficial role, reducing toxic events and promoting endogenous restorative processes [60,61].

The protection induced by MSCs is extremely variable across studies. In addition to methodological differences between injury models and laboratories, heterogeneity in MSC populations also contributes to disparate outcomes. MSCs obtained from different sources (for example, bone marrow versus umbilical cord blood versus amniotic fluid) are characterized by different protective potency. Furthermore, MSCs obtained from similar sources but from different donors can display different effects [62]. To predict *in vivo* potency, additional experimental work is needed to identify the specific properties of each MSC subpopulation, and to understand determinants of intrinsic heterogeneity. Before translating MSC-based therapies to the clinical environment, safety and consistency also need to be confirmed.

### Remote ischemic conditioning

Brief repeated cycles of peripheral vascular occlusion and de-occlusion in dogs prior to induction of coronary ischemia reduce myocardial infarct size [63]. This remote ischemic preconditioning is presumed to induce humoral factors that prevent reperfusion injury in several organs, including the brain. Protection occurs through modification of intracellular kinase activity, mitochondrial permeability and the inflammatory response to reperfusion [64]. One potential means by which ischemic conditioning can be achieved is by application of a standard blood pressure cuff to the arm and alternating 5-minute cycles of inflation and release [65].

Ischemic conditioning may be applied before (preconditioning), during (perconditioning) or after (postconditioning) a cerebral ischemic event. In patients, perconditioning as an adjunct to treatment with intravenous alteplase was associated with a reduction in tissue risk of infarction after acute thrombotic stroke [66]. Preconditioning has also been associated with prevention of recurrent stroke in patients with intracranial arterial stenosis [67]. Nevertheless, several questions remain unanswered: would remote ischemic conditioning reduce infarct size if administered before thrombolytic reperfusion of acute thrombotic stroke; can repeated (daily for weeks to months) ischemic conditioning improve long-term outcomes after cerebral ischemia; and in what other settings could ischemic conditioning reduce reperfusion injury and produce better clinical outcomes?

### Volatile anesthetic agents for neuroprotection

Volatile anesthetic agents may have neuroprotective properties. Pretreatment with isoflurane improved long-term neurological outcomes after experimental hypoxic/ischemic bran injury or focal brain ischemia [68] and post-treatment provided neuroprotection in rats [69]. The inducible form of nitric oxide synthase may mediate the tolerance to ischemia. Other factors that could be involved are the inhibition of excitatory neurotransmission and regulation of intracellular calcium responses during ischemia. Although attractive for their potential benefit on ischemic damage, these experiments have not been translated into clinical studies because use of a volatile agent may also induce vasodilatation, increasing CBF and, consequently, elevations in ICP.

In SAH patients, however, in whom an increase in CBF may be beneficial, the availability of a pragmatic bedside dispensing device and continuous ICP and regional CBF monitoring has made the inhalation of isoflurane for neuroprotection in the ICU practical, and this approach has been assessed in a small pilot study [70]. Nevertheless, at this stage, the use of volatile agents remains unproven and not ready for clinical implementation [71].

### Metabolic therapy: alternative fuel for the injured human brain

The brain can use alternative substrates beyond glucose, including lactate, pyruvate and ketone bodies, particularly in conditions of increased energy demand and limited glucose availability (for example, exercise, starvation, hypoglycemia or hypoxia/ischemia). Preferential use of lactate over glucose has been demonstrated in healthy human subjects [72] and diabetic patients [73]. Sodium lactate infusion has been shown to be neuroprotective in several brain injury models, both *in vitro* and *in vivo* [74-77]. Hypertonic solutions (containing sodium chloride or sodium lactate) may prevent brain edema and elevations of ICP following TBI [78,79]. Solutions containing lactate have favorable cerebral metabolic effects in patients with TBI [78]. Acetyl-L-carnitine infusion may also exert protective effects on the injured cells [80].

Overall, these data support the hypothesis that supplementation of the injured human brain with alternate energy fuels may be beneficial after acute brain injury [81]. Although a large trial of prehospital hypertonic saline infusions did not show beneficial effects on outcome [26], administration of isotonic or hypertonic solutions containing lactate, pyruvate or ketones may be a better option for the treatment of brain edema and cerebral ischemia following acute brain injury by enhancing brain energetics and, in turn, neurological recovery.

### Sex hormones

In numerous models, early parenteral administration of sex hormones has anti-apoptotic, anti-inflammatory and anti-oxidant properties and can accelerate reparative processes that prevent long-term sequelae [82]. One potential long-term reparative action of estrogens relates to their effects on sonic hedgehog, a signaling protein that controls and directs differentiation of neural stem cells, thus influencing brain repair by generating new neurons whenever necessary. Estrogen-induced acceleration of sonic hedgehog production may represent a potential pathway for neuroregeneration and neuroprotection [83]. Laboratory data have shown that the relevant effects of sex hormones in neurological emergencies, such as TBI, stroke and spinal cord injury, are neither cell-type specific nor insult specific [84,85].

Several clinical trials of sex hormones in TBI have recently been completed. The phase II RESCUE–TBI study (Clinical Trials NCT00973674) evaluated the safety and feasibility of administering a single dose of intravenous conjugated estrogens (Premarin; Pfizer Inc., New York, NY, USA) in patients with severe TBI, but no results are yet available. The National Institutes of Health-sponsored ProTECT III study evaluated the effects of intravenous progesterone (started within 4 hours of injury and given for a total of 96 hours) versus placebo in patients with moderate to severe TBI, but was stopped for futility [86]. The SyNAPSe Trial, comparing intravenous progesterone with placebo within 8 hours of severe TBI for a total of 120 hours, completed enrollment but failed to demonstrate a benefit [87]. There is therefore currently no clinical evidence to support sex hormone usage in TBI.

### Hyperoxia in neuroprotection

Oxygen is an essential substrate for the brain; however, the safety margin between effective and toxic oxygen doses is relatively narrow. Oxygen may be toxic to the lungs (for example, tracheobronchitis, absorption atelectasis, hypoxic pulmonary vasoconstriction and hyperoxia-induced lung injury), the circulation and the brain tissue itself (seizures or lipid peroxidation) [88], and hyperoxia has been associated with increased mortality in patients with various acute neurological disease processes [24,89-91]. However, hyperoxia can increase PbtO_2_, restore mitochondrial redox potential, decrease ICP, restore aerobic metabolism and improve pressure autoregulation [92-94]. Administering 100% oxygen at normal atmospheric pressure (normobaric hyperoxia) is inexpensive, widely available and can be started promptly after TBI or stroke (for example, by paramedics). Results in humans, however, have been mixed [24,93,95-97]. There is probably a narrow effective dose, and benefit may be limited to at-risk tissue. Moreover, treatment may only be effective in specific subgroups of patients or may depend on the metabolic state [97]. Furthermore, whether improvements in brain metabolism translate into better outcome is unclear [96].

Hyperbaric oxygen has been shown to reduce infarct volume, blood–brain barrier disruption, edema and neurologic deficits in animal models of ischemic brain injury [98]. In experimental TBI, hyperbaric oxygen decreased neuron injury and edema [99]. In a small group of severe TBI patients, hyperbaric oxygen improved brain metabolism and decreased ICP [100]. However, administering hyperbaric oxygen can be clinically challenging, requiring that patients are moved out of the ICU to the hyperbaric oxygen chamber, an expensive facility available in only a few centers. In a small study of 42 TBI patients, the combination of hyperbaric and normobaric hyperoxia was associated with significant outcome benefits compared with standard therapy [101]. These results need confirmation in larger studies.

PbtO_2_ monitoring is often used to optimize oxygenation targets and evaluate the utility of therapeutic interventions. Decreases in PbtO_2_ are associated with chemical markers of brain injury and with both mortality and unfavorable outcome after TBI [102], with similar (but less robust) associations in SAH. Addition of PbtO_2_-based care to conventional ICP-based and CPP-based care has been associated with improved outcomes after severe TBI [103,104]. This issue has been evaluated in a multicenter phase II clinical trial (Clinical Trials NCT00974259). Preliminary results demonstrate the feasibility and safety of PbtO_2_-based care and suggest a benefit to outcome, but phase III trials are necessary to confirm these findings.

## Lessons learned

Providing protection to the brain from different injuries has proven to be an ambitious goal, with a long series of clinical trial failures, especially for pharmacologic protection after TBI. In some instances, there may have been flaws in the experimental methodology (for example, relevance and quality of models, incomplete understanding of treatment mechanisms and inadequate elucidation of brain pharmacokinetics of the drug used). Many important methodological issues in animal studies are not adequately addressed before clinical trials are commenced (Box 1). It is often assumed that a disease mechanism detected in an animal model is also present in human disease, and that it has the same relevance, timing and duration as seen in the model. It is also often assumed that a candidate drug which is effective in reducing a pathophysiological process in animal models will have the same effect in humans. A careful analysis of failed neuroprotection trials suggests that these assumptions are unsafe [105]. The comprehensive monitoring of cerebral physiology and biochemistry commonly employed in critically ill patients with acute brain injury (especially TBI and SAH) provides a valuable opportunity to challenge some of these assumptions in preliminary studies, or as part of early phase II trials, before proceeding to more definitive studies (Box 2). Closer collaborations between preclinical investigators and clinical counterparts will be key to improving methodologies in both settings.

In clinical trials of TBI, outcome measures may not have been sufficiently sensitive and there has been a failure to adequately address the inherent heterogeneity of the patient populations [106]. The IMPACT studies have addressed methodological issues of trial design in TBI with an emphasis on dealing with the population heterogeneity and increasing sensitivity of outcome analysis [107] (Box 3). Although the IMPACT project has resulted in substantial advances by addressing problems related to prognostic heterogeneity, it did not address heterogeneity related to mechanism. Early mechanistic endpoints, which can serve as intermediate outcome markers in TBI trials, are still lacking. It will be impossible to mount a sufficient number of adequately powered clinical trials to address all existing uncertainties in the management of TBI. Alternative designs should therefore be considered. Rather than dealing with heterogeneity, we may be able to make use of it by employing comparative effectiveness research approaches [108].

Adaptive clinical trial design can reduce the risk of uninformative failed trials resulting from studies based on erroneous assumptions. Adaptive trials allow the preplanned modification of key clinical trial characteristics during the trial implementation phase based upon new information acquired within the trial itself and a set of carefully simulated rules (for example, changing the number of treatment arms, available dosing levels, randomization ratios and inclusion/exclusion criteria). The goal is to achieve statistical efficiency, improved ethical balance and scientific validity [109,110]. Compared with a traditional, fixed trial methodology, a well-designed adaptive trial can increase the probability of correctly identifying a truly effective therapy and can hasten the identification of futility, allowing scarce resources to be more rapidly directed towards the next promising target or project [111].

## Conclusion

**Figure 1:**
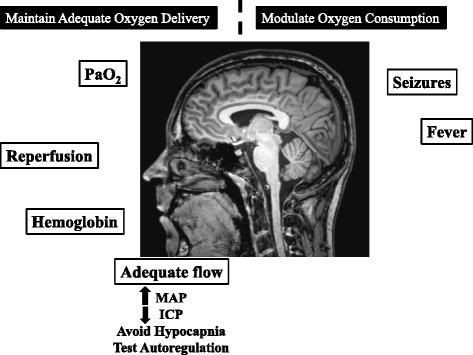
**Neuroprotective strategies in the ICU.** To avoid further insults to the brain (NMR image of a normal brain at the center), the goals of intensive care management are to ensure adequate oxygen delivery and to avoid excessive oxygen consumption, as in epileptic crises and cases of high brain temperature. See text for details on reperfusion after ischemic stroke, optimal hemoglobin level, and desirable cerebral perfusion pressure (CPP) levels. ICP, intracranial pressure; MAP, mean arterial pressure; PaO_2_, arterial oxygen tension.

Despite all the disappointments, there are many new therapeutic possibilities still to be explored and tested. Moreover, although the outcome benefits of specific agents and interventions have not been demonstrated, major advances in the clinical outcomes of our patients have been made in recent decades by applying a package of clinical measures, including meticulous monitoring and careful prevention and limitation of secondary insults in the early phases after injury (see Figure 1) [112].

### Box 1. Important aspects that need to be considered to improve preclinical studies in neuroprotection

1. Effects evaluated across injury severities.
2. Randomization and blinding procedures used.
3. Demonstrated effectiveness of structurally different drugs and genetic manipulation to confirm mechanism.
4. Evaluation of clinically relevant therapeutic window.
5. Examination of late functional outcomes as well as histological ones.
6. Elucidated pharmacokinetics and brain concentrations of drug associated with treatment efficacy.
7. Evaluation of treatment across sex and age spectrum.
8. Comparison of effects in multiple models and species.
9. Replication of therapeutic effect across laboratories.

### Box 2. Approaches to optimize translation of interventions from animal studies to clinical neuroprotection using experimental medicine approaches

1. Demonstration of existence, timing, and duration of target pathophysiology affected by candidate intervention in human disease (proof of mechanism).
2. Demonstration that the target molecule enters the brain in patients, at levels needed to produce benefit (favorable central nervous system pharmacokinetics).
3. Demonstration that the target molecule results in changes in target pathophysiological process in human disease (proof of concept).

### Box 3. Recommendations for clinical trial design from the International Mission on Prognosis and Analysis of randomized Controlled Trials in TBI study group

1. Inclusion criteria should be as broad as is compatible with the current understanding of the mechanism of action of the intervention being evaluated.
2. The statistical analysis should incorporate prespecified covariate adjustment to mitigate the effects of heterogeneity.
3. The statistical analysis should use an ordinal approach, based on either sliding dichotomy or proportional odds methodology.

## Abbreviations

CBF: Cerebral blood flow
CPP: Cerebral perfusion pressure
Hb: Hemoglobin
ICH: Intracerebral hemorrhage
ICP: Intracranial pressure
IMPACT: International Mission on Prognosis and Analysis of randomized Controlled Trials
MSC: Mesenchymal stromal cell
PbtO_2_: Brain tissue oxygen tension
SAH: Subarachnoid hemorrhage
TBI: Traumatic brain injury
TH: Therapeutic hypothermia

## References

[1] Selassie AW, Zaloshnja E, Langlois JA, Miller T, Jones P, Steiner C (2008). Incidence of long-term disability following traumatic brain injury hospitalization, United States, 2003. J Head Trauma Rehabil..

[2] Tagliaferri F, Compagnone C, Korsic M, Servadei F, Kraus J (2006). A systematic review of brain injury epidemiology in Europe. Acta Neurochir (Wien)..

[3] Roozenbeek B, Maas AI, Menon DK (2013). Changing patterns in the epidemiology of traumatic brain injury. Nat Rev Neurol..

[4] Hankey GJ (2013). The global and regional burden of stroke. Lancet Glob Health..

[5] Zacharia BE, Hickman ZL, Grobelny BT, DeRosa P, Kotchetkov I, Ducruet AF (2010). Epidemiology of aneurysmal subarachnoid hemorrhage. Neurosurg Clin N Am..

[6] Koenig MA (2014). Brain resuscitation and prognosis after cardiac arrest. Crit Care Clin..

[7] Barreto AD, Alexandrov AV (2012). Adjunctive and alternative approaches to current reperfusion therapy. Stroke..

[8] The National Institute of Neurological Disorders and Stroke rt-PA Stroke Study Group. Tissue plasminogen activator for acute ischemic stroke. N Engl J Med. 1995;333:1581–7. 10.1056/NEJM199512143332401 7477192

[9] Hacke W, Kaste M, Bluhmki E, Brozman M, Davalos A, Guidetti D (2008). Thrombolysis with alteplase 3 to 4.5 hours after acute ischemic stroke. N Engl J Med..

[10] Wardlaw JM, Murray V, Berge E, del Zoppo GJ (2014). Thrombolysis for acute ischaemic stroke. Cochrane Database Syst Rev..

[11] Jauch EC, Saver JL, Adams HP, Bruno A, Connors JJ, Demaerschalk BM (2013). Guidelines for the early management of patients with acute ischemic stroke: a guideline for healthcare professionals from the American Heart Association/American Stroke Association. Stroke..

[12] Lansberg MG, Straka M, Kemp S, Mlynash M, Wechsler LR, Jovin TG (2012). MRI profile and response to endovascular reperfusion after stroke (DEFUSE 2): a prospective cohort study. Lancet Neurol..

[13] Kidwell CS, Jahan R, Gornbein J, Alger JR, Nenov V, Ajani Z (2013). A trial of imaging selection and endovascular treatment for ischemic stroke. N Engl J Med..

[14] Lee M, Hong KS, Saver JL (2010). Efficacy of intra-arterial fibrinolysis for acute ischemic stroke: meta-analysis of randomized controlled trials. Stroke..

[15] Broderick JP, Palesch YY, Demchuk AM, Yeatts SD, Khatri P, Hill MD (2013). Endovascular therapy after intravenous t-PA versus t-PA alone for stroke. N Engl J Med..

[16] Ciccone A, Valvassori L, Nichelatti M, Sgoifo A, Ponzio M, Sterzi R (2013). Endovascular treatment for acute ischemic stroke. N Engl J Med..

[17] Berkhemer OA, Fransen PS, Beumer D, van den Berg LA, Lingsma HF, Yoo AJ (2015). A randomized trial of intraarterial treatment for acute ischemic stroke. N Engl J Med..

[18] Nelson KS, Brearley AM, Haines SJ (2014). Evidence-based assessment of well-established interventions: the parachute and the epidural hematoma. Neurosurgery..

[19] Bricolo AP, Pasut LM (1984). Extradural hematoma: toward zero mortality. A prospective study. Neurosurgery..

[20] Cooper DJ, Rosenfeld JV, Murray L, Arabi YM, Davies AR, D'Urso P (2011). Decompressive craniectomy in diffuse traumatic brain injury. N Engl J Med..

[21] Chesnut RM, Marshall LF, Klauber MR, Blunt BA, Baldwin N, Eisenberg HM (1993). The role of secondary brain injury in determining outcome from severe head injury. J Trauma..

[22] McHugh GS, Engel DC, Butcher I, Steyerberg EW, Lu J, Mushkudiani N (2007). Prognostic value of secondary insults in traumatic brain injury: results from the IMPACT study. J Neurotrauma..

[23] Franschman G, Peerdeman SM, Andriessen TM, Greuters S, Toor AE, Vos PE (2011). Effect of secondary prehospital risk factors on outcome in severe traumatic brain injury in the context of fast access to trauma care. J Trauma..

[24] Brenner M, Stein D, Hu P, Kufera J, Wooford M, Scalea T (2012). Association between early hyperoxia and worse outcomes after traumatic brain injury. Arch Surg..

[25] Bratton SL, Chestnut RM, Ghajar J, McConnell Hammond FF, Harris OA, Hartl R, et al. Guidelines for the management of severe traumatic brain injury. I. Blood pressure and oxygenation. J Neurotrauma. 2007;24 Suppl 1:S7-13. 10.1089/neu.2007.9995 17511549

[26] Bulger EM, May S, Brasel KJ, Schreiber M, Kerby JD, Tisherman SA (2010). Out-of-hospital hypertonic resuscitation following severe traumatic brain injury: a randomized controlled trial. JAMA..

[27] Wang HE, Peitzman AB, Cassidy LD, Adelson PD, Yealy DM (2004). Out-of-hospital endotracheal intubation and outcome after traumatic brain injury. Ann Emerg Med..

[28] Bernard SA, Nguyen V, Cameron P, Masci K, Fitzgerald M, Cooper DJ (2010). Prehospital rapid sequence intubation improves functional outcome for patients with severe traumatic brain injury: a randomized controlled trial. Ann Surg..

[29] Stein SC, Georgoff P, Meghan S, Mirza KL, El Falaky OM (2010). Relationship of aggressive monitoring and treatment to improved outcomes in severe traumatic brain injury. J Neurosurg..

[30] Rangel-Castilla L, Gasco J, Nauta HJ, Okonkwo DO, Robertson CS (2008). Cerebral pressure autoregulation in traumatic brain injury. Neurosurg Focus..

[31] Steiner LA, Czosnyka M, Piechnik SK, Smielewski P, Chatfield D, Menon DK (2002). Continuous monitoring of cerebrovascular pressure reactivity allows determination of optimal cerebral perfusion pressure in patients with traumatic brain injury. Crit Care Med..

[32] LeRoux P (2013). Haemoglobin management in acute brain injury. Curr Opin Crit Care..

[33] Kramer AH, Zygun DA (2009). Anemia and red blood cell transfusion in neurocritical care. Crit Care..

[34] Retter A, Wyncoll D, Pearse R, Carson D, McKechnie S, Stanworth S (2013). Guidelines on the management of anaemia and red cell transfusion in adult critically ill patients. Br J Haematol..

[35] Hebert PC, Wells G, Blajchman MA, Marshall J, Martin C, Pagliarello G (1999). A multicenter, randomized, controlled clinical trial of transfusion requirements in critical care. Transfusion requirements in critical care investigators, Canadian critical care trials group. N Engl J Med.

[36] Le Roux PD (2011). Anemia and transfusion after subarachnoid hemorrhage. Neurocrit Care..

[37] Robertson CS, Hannay HJ, Yamal JM, Gopinath S, Goodman JC, Tilley BC (2014). Effect of erythropoietin and transfusion threshold on neurological recovery after traumatic brain injury: a randomized clinical trial. JAMA..

[38] Naidech AM, Shaibani A, Garg RK, Duran IM, Liebling SM, Bassin SL (2010). Prospective, randomized trial of higher goal hemoglobin after subarachnoid hemorrhage. Neurocrit Care..

[39] Kumar MA, Boland TA, Baiou M, Moussouttas M, Herman JH, Bell RD (2014). Red blood cell transfusion increases the risk of thrombotic events in patients with subarachnoid hemorrhage. Neurocrit Care..

[40] Oddo M, Levine JM, Kumar M, Iglesias K, Frangos S, Maloney-Wilensky E (2012). Anemia and brain oxygen after severe traumatic brain injury. Intensive Care Med..

[41] Sonneville R, Verdonk F, Rauturier C, Klein IF, Wolff M, Annane D (2013). Understanding brain dysfunction in sepsis. Ann Intensive Care..

[42] Taccone FS, Scolletta S, Franchi F, Donadello K, Oddo M (2013). Brain perfusion in sepsis. Curr Vasc Pharmacol..

[43] Schramm P, Klein KU, Falkenberg L, Berres M, Closhen D, Werhahn KJ (2012). Impaired cerebrovascular autoregulation in patients with severe sepsis and sepsis-associated delirium. Crit Care..

[44] Hughes CG, Morandi A, Girard TD, Riedel B, Thompson JL, Shintani AK (2013). Association between endothelial dysfunction and acute brain dysfunction during critical illness. Anesthesiology..

[45] Taccone FS, Su F, Pierrakos C, He X, James S, Dewitte O (2010). Cerebral microcirculation is impaired during sepsis: an experimental study. Crit Care..

[46] Bemeur C, Butterworth RF (2013). Liver–brain proinflammatory signalling in acute liver failure: role in the pathogenesis of hepatic encephalopathy and brain edema. Metab Brain Dis..

[47] Ytrebo LM, Kristiansen RG, Maehre H, Fuskevag OM, Kalstad T, Revhaug A (2009). L-ornithine phenylacetate attenuates increased arterial and extracellular brain ammonia and prevents intracranial hypertension in pigs with acute liver failure. Hepatology..

[48] Jalan R, Olde Damink SW, Deutz NE, Davies NA, Garden OJ, Madhavan KK (2003). Moderate hypothermia prevents cerebral hyperemia and increase in intracranial pressure in patients undergoing liver transplantation for acute liver failure. Transplantation..

[49] Murphy N, Auzinger G, Bernel W, Wendon J (2004). The effect of hypertonic sodium chloride on intracranial pressure in patients with acute liver failure. Hepatology..

[50] Rossi S, Zanier ER, Mauri I, Columbo A, Stocchetti N (2001). Brain temperature, body core temperature, and intracranial pressure in acute cerebral damage. J Neurol Neurosurg Psychiatry..

[51] Polderman KH (2008). Induced hypothermia and fever control for prevention and treatment of neurological injuries. Lancet..

[52] Polderman KH (2009). Mechanisms of action, physiological effects, and complications of hypothermia. Crit Care Med..

[53] Yenari MA, Han HS (2012). Neuroprotective mechanisms of hypothermia in brain ischaemia. Nat Rev Neurosci..

[54] Nielsen N, Wetterslev J, Cronberg T, Erlinge D, Gasche Y, Hassager C (2013). Targeted temperature management at 33 degrees C versus 36 degrees C after cardiac arrest. N Engl J Med..

[55] Varon J, Polderman K (2014). Targeted temperature management after cardiac arrest. N Engl J Med..

[56] Wu X, Drabek T, Kochanek PM, Henchir J, Stezoski SW, Stezoski J (2006). Induction of profound hypothermia for emergency preservation and resuscitation allows intact survival after cardiac arrest resulting from prolonged lethal hemorrhage and trauma in dogs. Circulation..

[57] Wu X, Drabek T, Tisherman SA, Henchir J, Stezoski SW, Culver S (2008). Emergency preservation and resuscitation with profound hypothermia, oxygen, and glucose allows reliable neurological recovery after 3 h of cardiac arrest from rapid exsanguination in dogs. J Cereb Blood Flow Metab..

[58] Lo EH (2008). A new penumbra: transitioning from injury into repair after stroke. Nat Med..

[59] Laroni A, Novi G, de Kerlero RN, Uccelli A (2013). Towards clinical application of mesenchymal stem cells for treatment of neurological diseases of the central nervous system. J Neuroimmune Pharmacol..

[60] Phinney DG, Sensebe L (2013). Mesenchymal stromal cells: misconceptions and evolving concepts. Cytotherapy..

[61] Zanier ER, Pischiutta F, Riganti L, Marchesi F, Turola E, Fumagalli S (2014). Bone marrow mesenchymal stromal cells drive protective m2 microglia polarization after brain trauma. Neurotherapeutics..

[62] Zanier ER, Montinaro M, Vigano M, Villa P, Fumagalli S, Pischiutta F (2011). Human umbilical cord blood mesenchymal stem cells protect mice brain after trauma. Crit Care Med..

[63] Murry CE, Jennings RB, Reimer KA (1986). Preconditioning with ischemia: a delay of lethal cell injury in ischemic myocardium. Circulation..

[64] Kharbanda RK, Nielsen TT, Redington AN (2009). Translation of remote ischaemic preconditioning into clinical practice. Lancet..

[65] Kharbanda RK, Mortensen UM, White PA, Kristiansen SB, Schmidt MR, Hoschtitzky JA (2002). Transient limb ischemia induces remote ischemic preconditioning in vivo. Circulation..

[66] Hougaard KD, Hjort N, Zeidler D, Sorensen L, Norgaard A, Hansen TM (2014). Remote ischemic perconditioning as an adjunct therapy to thrombolysis in patients with acute ischemic stroke: a randomized trial. Stroke..

[67] Meng R, Asmaro K, Meng L, Liu Y, Ma C, Xi C (2012). Upper limb ischemic preconditioning prevents recurrent stroke in intracranial arterial stenosis. Neurology..

[68] McAuliffe JJ, Joseph B, Vorhees CV (2007). Isoflurane-delayed preconditioning reduces immediate mortality and improves striatal function in adult mice after neonatal hypoxia-ischemia. Anesth Analg..

[69] Segal N, Matsuura T, Caldwell E, Sarraf M, McKnite S, Zviman M (2012). Ischemic postconditioning at the initiation of cardiopulmonary resuscitation facilitates functional cardiac and cerebral recovery after prolonged untreated ventricular fibrillation. Resuscitation..

[70] Villa F, Iacca C, Molinari AF, Giussani C, Aletti G, Pesenti A (2012). Inhalation versus endovenous sedation in subarachnoid hemorrhage patients: effects on regional cerebral blood flow. Crit Care Med..

[71] Bosel J, Purrucker JC, Nowak F, Renzland J, Schiller P, Perez EB (2012). Volatile isoflurane sedation in cerebrovascular intensive care patients using AnaConDa(®): effects on cerebral oxygenation, circulation, and pressure. Intensive Care Med..

[72] Smith D, Pernet A, Hallett WA, Bingham E, Marsden PK, Amiel SA (2003). Lactate: a preferred fuel for human brain metabolism in vivo. J Cereb Blood Flow Metab..

[73] Maran A, Cranston I, Lomas J, Macdonald I, Amiel SA (1994). Protection by lactate of cerebral function during hypoglycaemia. Lancet..

[74] Berthet C, Castillo X, Magistretti PJ, Hirt L (2012). New evidence of neuroprotection by lactate after transient focal cerebral ischaemia: extended benefit after intracerebroventricular injection and efficacy of intravenous administration. Cerebrovasc Dis..

[75] Moxon-Lester L, Sinclair K, Burke C, Cowin GJ, Rose SE, Colditz P (2007). Increased cerebral lactate during hypoxia may be neuroprotective in newborn piglets with intrauterine growth restriction. Brain Res..

[76] Herzog RI, Jiang L, Herman P, Zhao C, Sanganahalli BG, Mason GF (2013). Lactate preserves neuronal metabolism and function following antecedent recurrent hypoglycemia. J Clin Invest..

[77] Rice AC, Zsoldos R, Chen T, Wilson MS, Alessandri B, Hamm RJ (2002). Lactate administration attenuates cognitive deficits following traumatic brain injury. Brain Res..

[78] Bouzat P, Sala N, Suys T, Zerlauth JB, Marques-Vidal P, Feihl F (2014). Cerebral metabolic effects of exogenous lactate supplementation on the injured human brain. Intensive Care Med..

[79] Ichai C, Payen JF, Orban JC, Quintard H, Roth H, Legrand R (2013). Half-molar sodium lactate infusion to prevent intracranial hypertensive episodes in severe traumatic brain injured patients: a randomized controlled trial. Intensive Care Med..

[80] Patel SP, Sullivan PG, Lyttle TS, Magnuson DS, Rabchevsky AG (2012). Acetyl-L-carnitine treatment following spinal cord injury improves mitochondrial function correlated with remarkable tissue sparing and functional recovery. Neuroscience..

[81] White H, Venkatesh B, Jones M, Worrall S, Chuah T, Ordonez J (2013). Effect of a hypertonic balanced ketone solution on plasma, CSF and brain beta-hydroxybutyrate levels and acid–base status. Intensive Care Med..

[82] Gatson JW, Warren V, AbdelFattah K, Wolf S, Hynan LS, Moore C (2013). Detection of beta-amyloid oligomers as a predictor of neurological outcome after brain injury. J Neurosurg..

[83] Pepe PE, Wigginton JG, Gatson JW, Simpkins JW, Maass DL, AbdelFattah K, et al. Single dose estrogen infusion can amplify brain levels of sonic hedgehog (SHH), a signal protein for neuro stem cells and repair following the indirect brain injury resulting after severe torso burns [abstract]. Crit Care. 2013;17 Suppl 2:P287.

[84] Simpkins JW, Yi KD, Yang SH, Dykens JA (2010). Mitochondrial mechanisms of estrogen neuroprotection. Biochim Biophys Acta..

[85] Sayeed I, Stein DG (2009). Progesterone as a neuroprotective factor in traumatic and ischemic brain injury. Prog Brain Res..

[86] Wright DW, Yeatts SD, Silbergleit R, Palesch YY, Hertzberg VS, Frankel M (2014). Very early administration of progesterone for acute traumatic brain injury. N Engl J Med..

[87] Skolnick BE, Maas AI, Narayan RK, van der Hoop RG, MacAllister T, Ward JD (2014). A clinical trial of progesterone for severe traumatic brain injury. N Engl J Med..

[88] Lambertsen CJ, Dough RH, Cooper DJ, Emmel GL, Loeschcke HH, Schmidt CF (1953). Oxygen toxicity: effects in man of oxygen inhalation at 1 and 3.5 atmospheres upon blood gas transport, cerebral circulation and cerebral metabolism. J Appl Physiol.

[89] Rincon F, Kang J, Maltenfort M, Vibbert M, Urtecho J, Athar MK (2014). Association between hyperoxia and mortality after stroke: a multicenter cohort study. Crit Care Med..

[90] Kilgannon JH, Jones AE, Shapiro NI, Angelos MG, Milcarek B, Hunter K (2010). Association between arterial hyperoxia following resuscitation from cardiac arrest and in-hospital mortality. JAMA..

[91] Rincon F, Kang J, Vibbert M, Urtecho J, Athar MK, Jallo J (2014). Significance of arterial hyperoxia and relationship with case fatality in traumatic brain injury: a multicentre cohort study. J Neurol Neurosurg Psychiatry..

[92] Rockswold SB, Rockswold GL, Zaun DA, Zhang X, Cerra CE, Bergman TA (2010). A prospective, randomized clinical trial to compare the effect of hyperbaric to normobaric hyperoxia on cerebral metabolism, intracranial pressure, and oxygen toxicity in severe traumatic brain injury. J Neurosurg..

[93] Tolias CM, Reinert M, Seiler R, Gilman C, Scharf A, Bullock MR (2004). Normobaric hyperoxia-induced improvement in cerebral metabolism and reduction in intracranial pressure in patients with severe head injury: a prospective historical cohort-matched study. J Neurosurg..

[94] Nortje J, Coles JP, Timofeev I, Fryer TD, Aigbirhio FI, Smielewski P (2008). Effect of hyperoxia on regional oxygenation and metabolism after severe traumatic brain injury: preliminary findings. Crit Care Med..

[95] Diringer MN, Aiyagari V, Zazulia AR, Videen TO, Powers WJ (2007). Effect of hyperoxia on cerebral metabolic rate for oxygen measured using positron emission tomography in patients with acute severe head injury. J Neurosurg..

[96] Quintard H, Patet C, Suys T, Marques-Vidal P, Oddo M (2014). Normobaric hyperoxia is associated with increased cerebral excitotoxicity after severe traumatic brain injury. Neurocrit Care. Epub ahead of print.

[97] Vilalta A, Sahuquillo J, Merino MA, Poca MA, Garnacho A, Martinez-Valverde T (2011). Normobaric hyperoxia in traumatic brain injury: does brain metabolic state influence the response to hyperoxic challenge?. J Neurotrauma..

[98] Miljkovic-Lolic M, Silbergleit R, Fiskum G, Rosenthal RE (2003). Neuroprotective effects of hyperbaric oxygen treatment in experimental focal cerebral ischemia are associated with reduced brain leukocyte myeloperoxidase activity. Brain Res..

[99] Vlodavsky E, Palzur E, Soustiel JF (2006). Hyperbaric oxygen therapy reduces neuroinflammation and expression of matrix metalloproteinase-9 in the rat model of traumatic brain injury. Neuropathol Appl Neurobiol..

[100] Rockswold SB, Rockswold GL, Vargo JM, Erickson CA, Sutton RL, Bergman TA (2001). Effects of hyperbaric oxygenation therapy on cerebral metabolism and intracranial pressure in severely brain injured patients. J Neurosurg..

[101] Rockswold SB, Rockswold GL, Zaun DA, Liu J (2013). A prospective, randomized phase II clinical trial to evaluate the effect of combined hyperbaric and normobaric hyperoxia on cerebral metabolism, intracranial pressure, oxygen toxicity, and clinical outcome in severe traumatic brain injury. J Neurosurg..

[102] Oddo M, Levine JM, Mackenzie L, Frangos S, Feihl F, Kasner SE (2011). Brain hypoxia is associated with short-term outcome after severe traumatic brain injury independently of intracranial hypertension and low cerebral perfusion pressure. Neurosurgery..

[103] Spiotta AM, Stiefel MF, Gracias VH, Garuffe AM, Kofke WA, Maloney-Wilensky E (2010). Brain tissue oxygen-directed management and outcome in patients with severe traumatic brain injury. J Neurosurg..

[104] Narotam PK, Morrison JF, Nathoo N (2009). Brain tissue oxygen monitoring in traumatic brain injury and major trauma: outcome analysis of a brain tissue oxygen-directed therapy. J Neurosurg..

[105] Janowitz T, Menon DK. Exploring new routes for neuroprotective drug development in traumatic brain injury. Sci Transl Med. 2010;2:27rv1. 10.1126/scitranslmed.3000330 20393189

[106] Loane DJ, Faden AI (2010). Neuroprotection for traumatic brain injury: translational challenges and emerging therapeutic strategies. Trends Pharmacol Sci..

[107] Maas AI, Steyerberg EW, Marmarou A, McHugh GS, Lingsma HF, Butcher I (2010). IMPACT recommendations for improving the design and analysis of clinical trials in moderate to severe traumatic brain injury. Neurotherapeutics..

[108] Maas AI, Menon DK, Lingsma HF, Pineda JA, Sandel ME, Manley GT (2012). Re-orientation of clinical research in traumatic brain injury: report of an international workshop on comparative effectiveness research. J Neurotrauma..

[109] Berry DA (2006). Bayesian clinical trials. Nat Rev Drug Discov..

[110] Hung HM, O'Neill RT, Wang SJ, Lawrence J (2006). A regulatory view on adaptive/flexible clinical trial design. Biom J..

[111] Dragalin V (2006). Adaptive designs: terminology and classification. Drug Informat..

[112] Stein SC, Georgoff P, Meghan S, Mizra K, Sonnad SS (2010). 150 years of treating severe traumatic brain injury: a systematic review of progress in mortality. J Neurotrauma..

